# Supplementary material for: Rapid and robust assembly and decoding of molecular tags with DNA-based nanopore signatures
Source: Nat Commun. 2020 Nov 3;11:5454. doi: 10.1038/s41467-020-19151-8 (PMC7642340; doi:10.1038/s41467-020-19151-8)
Supplement: Supplementary file 1 — Supplementary Information [file 41467_2020_19151_MOESM1_ESM.pdf]

Supplementary Information for: Rapid and robust assembly and decoding of molecular tags with DNA-based nanopore signatures

Doroschak et al.

Supplementary Methods

Evaluating read count variation

As described in the main text, the largest source of bit errors in Porcupine can be attributed to the unusually large but seemingly consistent read count variance. Since each molecular tag is composed of equal proportions of each present molbit, the resulting read counts for each molbit after sequencing should be approximately equal; but instead, we measured significant variation in read counts. We first checked for obvious sequence-related issues by correlating quantifiable sequence characteristics, including GC content, minimum free energy (MFE), and homopolymer length, against read counts normalized as described in the main text. Evidence shows GC bias in genomic bacterial nanopore sequencing data on the MinION [1], based on non-stochastic bias introduced by the basecaller, not the sequencing device. With respect to MFE, we reasoned that although we controlled for MFE in the design phase, correlation between folding energy and read counts could still explain some variation. Additionally, we considered that the inclusion of long homopolymers could also have been associated with a reduction in read counts since imprecise calls of homopolymer length can potentially cause poor sequence alignment and therefore artificially low read counts [2]. We also checked for occurrences of the 4-base overhangs used for Golden Gate assembly (GAGT and GCTG), and found no notable trends. As shown in Supplementary Figure 8a, correlations between these various measures and normalized read counts were quite low (Pearson correlation (two-sided) for GC: $p = 0.31$, $r=-0.10$; MFE: $p=0.33$, $r=0.09$; homopolymer length: $p=0.82$, $r=0.02$; GCTG presence: $p=0.90$, $r=0.01$; GAGT presence: $p=0.42$, $r=-0.08$). Thus, these rough measures could not explain the source of read count variance.

Moving beyond these initial sequence bias analyses, we reasoned that read count variation errors can be introduced at four different points in our system: (1) DNA synthesis, (2) strand

assembly and tag combination, (3) sequencing, or (4) analysis/labeling.

(1) To check whether there was a problem with our original DNA order, we re-ordered 15 molbits representing the five lowest, average, and highest read count molbits. When sequenced, the read counts of the re-ordered sequences were similar in proportion to their previous counts (Supplementary Figure 8b). Thus, if the source of variability is caused by a synthesis error, which we believe is unlikely, it is at least reproducible or possibly dependent on an unexplored aspect of sequence content.

(2) To evaluate the strand assembly and tag combination step, we examined potential secondary structure in individual molbits, specifically the same molbits from the re-synthesized tests. During the Golden Gate strand assembly step, double stranded DNA is separated into single strands, potentially enabling secondary structure formation. Sequences were screened for minimum free energy during the evolutionary modeling phase, but the screening did not cover all forms of secondary structure, particularly stem-loop structures with short stems and large loops. Some secondary structure can be seen across all three categories (see Supplementary Figure 9a). It is possible that secondary structure could have lowered read counts, but we do not believe that it could have caused excessive read counts.

Additionally, we reasoned about potential experimental variation due to human error. When combining tags, some variation can be explained by pipetting. Here, we reasoned that up to 2-3x variation could be explained by this factor (bubbles, calibration, improperly depressed pipette, etc.). Also, if a bit were omitted, it would cause a near-zero or very low read count, but if a bit were accidentally added, it alone would not cause such a dramatic overabundance of read counts. Thus, human error of this type could cause a low read count but likely not an overly high one.

(3) It is possible the systematic sequencing errors contribute to molbit read differences, however, the strong correlation between basecalling and CNN-based molbit classification makes this less likely. Future experiments using a different sequencing platform (e.g. Illumina or Sanger), could help to resolve this question.

(4) We do not believe basecalling or labeling is a significant contributor to the problem. With respect to basecalling, an average of 93.3% of reads could be basecalled with high confidence (Q-score ≥ 9) throughout all runs, leaving just $\sim$7% room for variation (compared to 200-300% variation overall).

Also note that the molbit counts for labels assigned via basecalling and alignment have high correlation with those assigned by the CNN (Pearson two-sided $p < 10^{-5}$, r=0.9998, Supplementary Figure 2. Although there is certainly bias in that figure since basecalled labels are used to train the CNN, this high correlation does reduce the possibility that either method is introducing a significant number of spurious calls.

Ultimately, our method was designed to be robust to these types of errors; however, we still

sought to reduce them since any reduction in bit errors can exponentially reduce the overall decoding error rate.

Length encoding

The threshold for determining length was chosen by finding the point that maximized the number of correctly assigned reads when comparing separate runs containing 400 or 1600 bp long sequences. For the 1600 bp reads that fell below this threshold, we examined their sequence contents for evidence of fragmentation. The majority of the short strands in the 1600 bp sample can be explained by fragmentation, supported by an evaluation of basecalled, aligned sequences. After aligning all basecalled sequences to the insert fragment sequence using BWA-MEM [3], we examined reads that were shorter than the signal length threshold (i.e., those mislabeled as 400 bp). We found that 28% of these reads were truncated, meaning they aligned well to the beginning of the insert but terminated prematurely at various lengths. Another 59% mapped the majority of the read to a random portion of the reference, indicating fragmentation. The remaining reads were simply poor quality reads that did not basecall and/or map well. To mitigate this, a length purification step could be added, making preparation longer to provide a higher quality readout.

Error correction

To improve the system's resilience we overlay an error correcting code. The error correcting code (ECC) maps the original messages into a higher dimensional space that provides a greater distance between any two messages. In this higher dimensional space a message must accumulate many errors before it is decoded incorrectly, allowing us to reduce the chance of incorrect decoding for a given fixed error rate.

The error correcting code protocol has two stages: encoding and decoding. We use a random linear code, which consists of a fixed, randomly chosen $n \times 96$ generator matrix that encodes an n-bit message via a simple and efficient matrix multiplication. The resulting 96-molbit codeword may accumulate errors during creation, storage, or retrieval. However, we can decode the 96-molbit codeword back to the n-bit message with high probability using brute-force nearest neighbor decoding.

We also computationally examined whether limiting the set of molbits to only the most performant would improve overall tag accuracy. Unfortunately, as the total number of molbits drops, they must each become more reliable in order to maintain the desired chance of incorrect decoding of 10^{-9} (Supplementary Figure 10). Another way to think about this tradeoff is to consider that even unreliable molbits are still conveying useful information that the ECC is able to use to deduce the corrected message. For example, a collection of 96 molbits can reliably

encode a 32-bit message even if each molbit is wrong 3% of the time on average. But if we reduce our pool slightly to just 90 molbits, then they can only be wrong 2% of the time on average. At 64 molbits, a dramatic reduction in the corruption rate would be required, down to the order of 0.15%.

Supplementary Table

Primer name	Sequence
400mer_F	GCCGGTCTCTGCTGCTTTAATAGTGGACTCTTGTTCCAAACTGG
400mer_R	GCCGGTCTCTGCTGTACCAGGATCTTGCCATCCTATGGAAC
1600mer_F	GCCGGTCTCTGCTGGATCCTTTTTTTCTGCGCGTAATCTGC
1600mer_R	GCCGGTCTCTGCTGGAATGAATCACCGATACGCGAGCG

Table 1: **Primer sequences used to extract the insert strand from plasmid pCDB180 by PCR (see Methods).**

Supplementary Figures 1-10

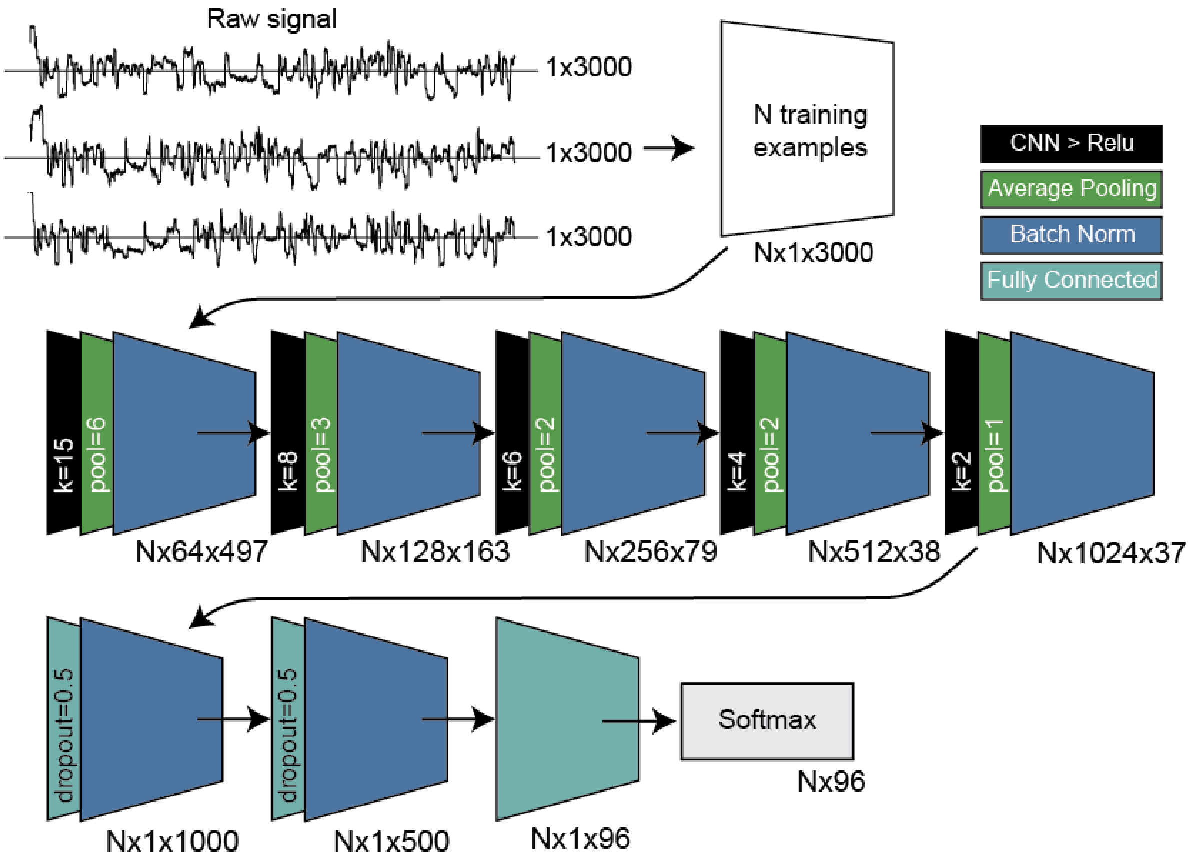

Supplementary Figure 1: Molbit classification model and data flow. Input data includes raw nanopore signal, which was rescaled using a Median Absolute Deviation method modified from Mako, trimmed to remove stalled signal characteristic to the beginning of sequencing reads, and truncated to the first 3000 data points in the signal time series. Rescaled training data then passes through a 5-layer CNN followed by 2 fully connected layers with dropout and a final fully connected layer with softmax as the output layer. In the CNN layers, k is the kernel size and pool is the average pooling kernel size.

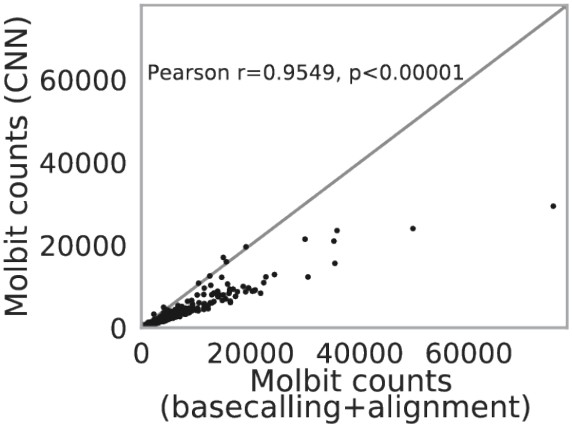

Supplementary Figure 2: Comparison of molbit counts from sequence data (labeled using base-calling plus alignment) vs. signal data (labeled using CNN).

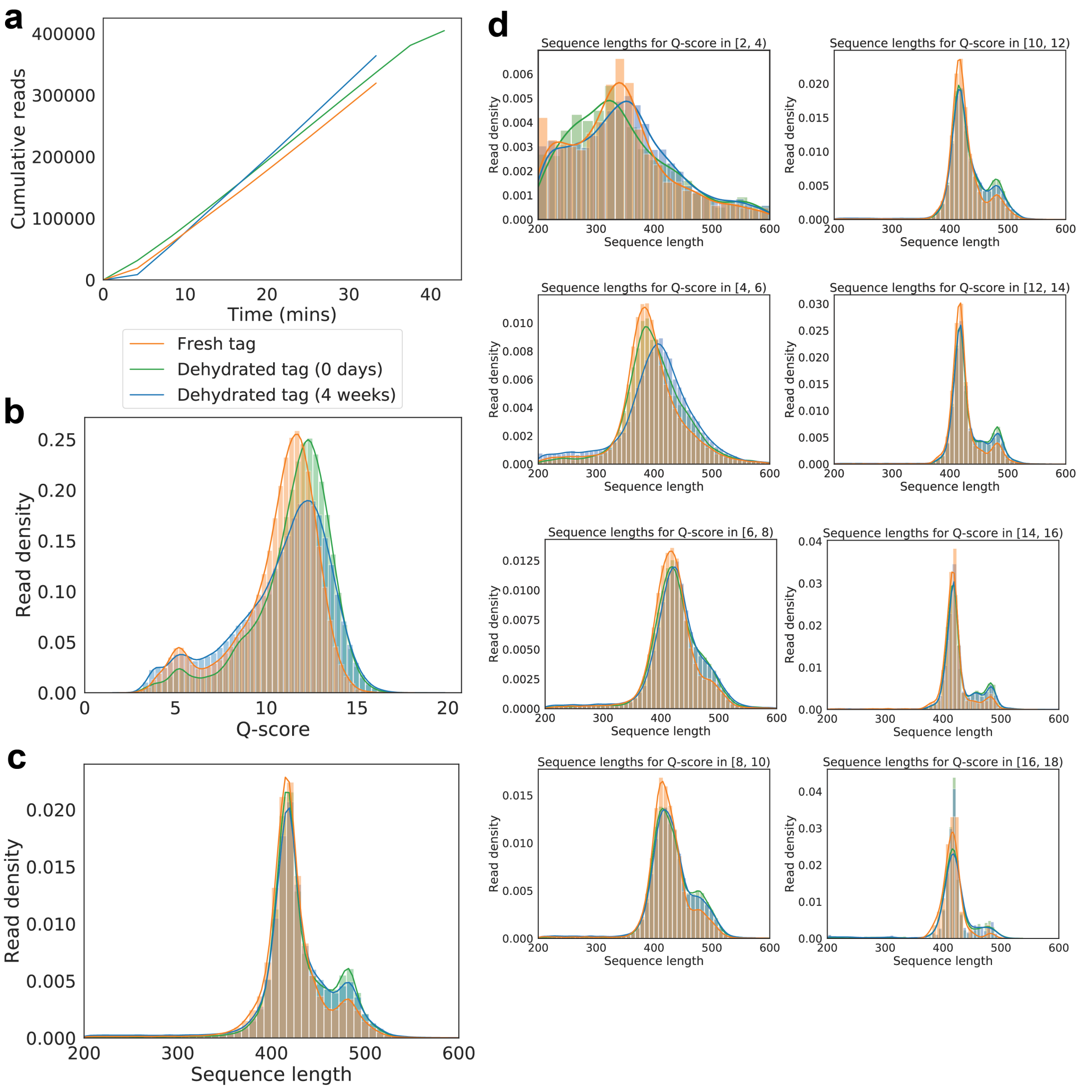

Supplementary Figure 3: Quality of fresh versus dehydrated tags. The fresh and dehydrated tag libraries were prepared at separate times, and the dehydrated tags were prepared as one library, then split after dehydration. (a) Cumulative sequencing read output over time. (b) Distribution of PHRED quality scores for all reads after basecalling using Guppy 3.2.2 (GPU version). (c) Distribution of sequence lengths after basecalling. (d) Same as (c) but split in bands of Q-scores.

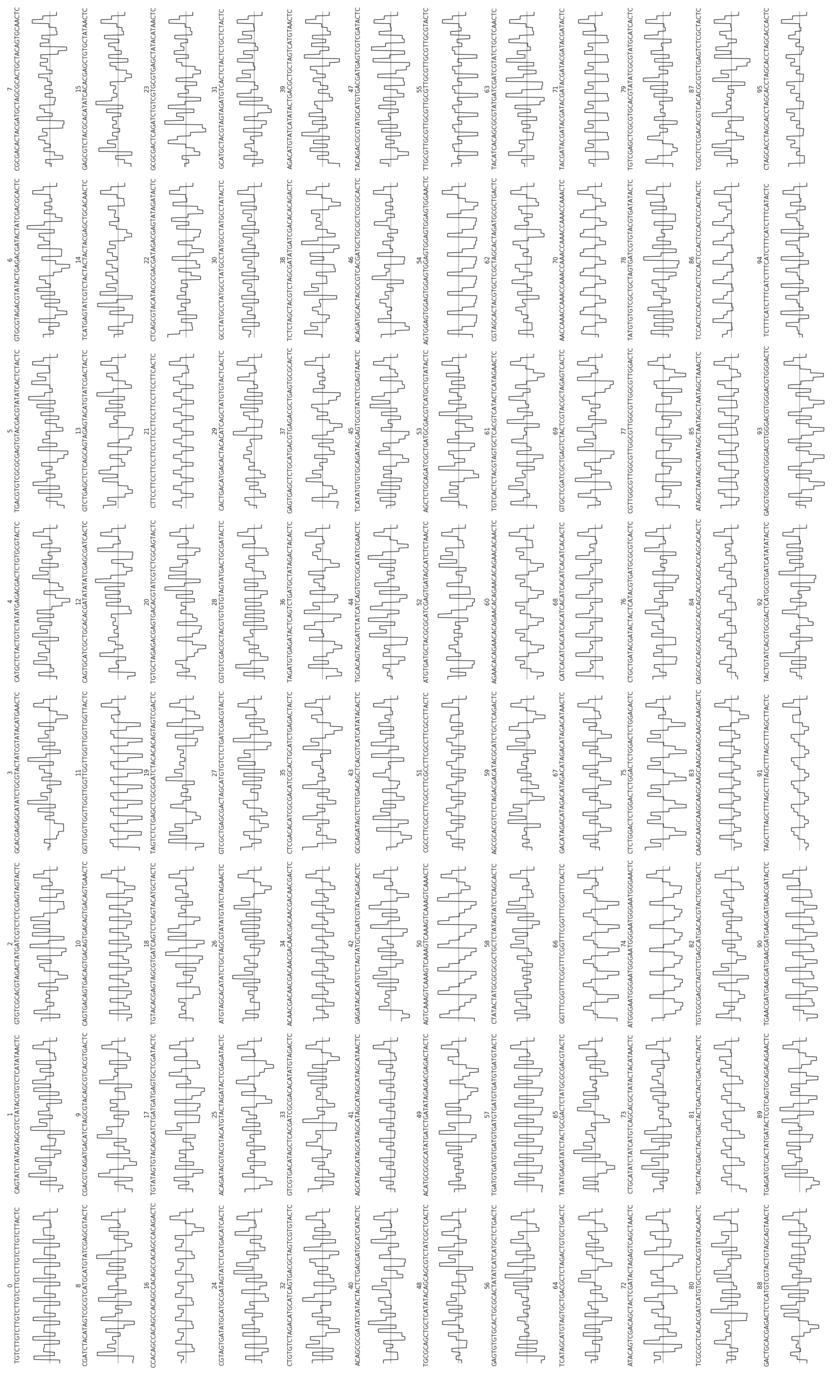

Supplementary Figure 4: All 96 molbit sequences initialized before beginning the design phase using the evolutionary model.

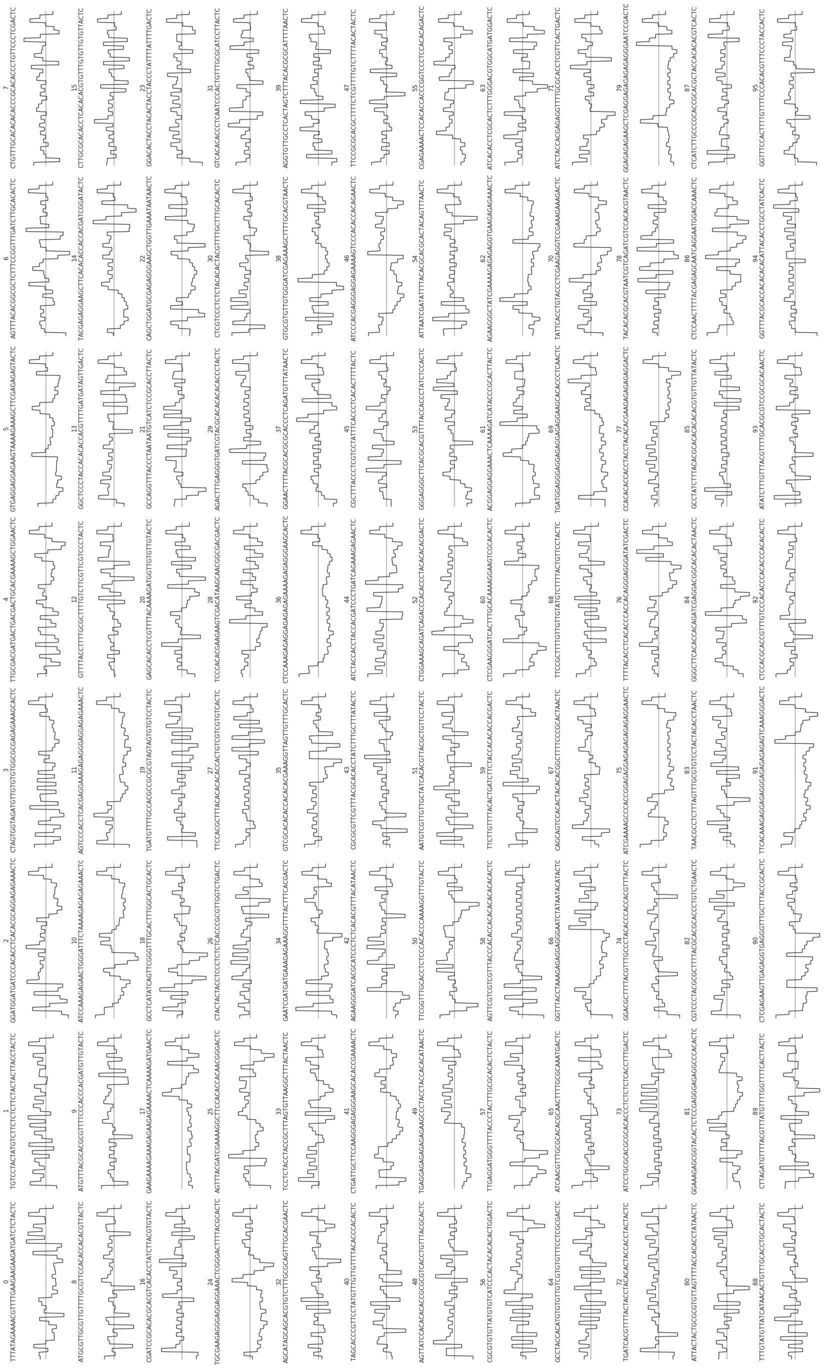

Supplementary Figure 5: All 96 molbit sequences after 31 iterations of the evolutionary model.

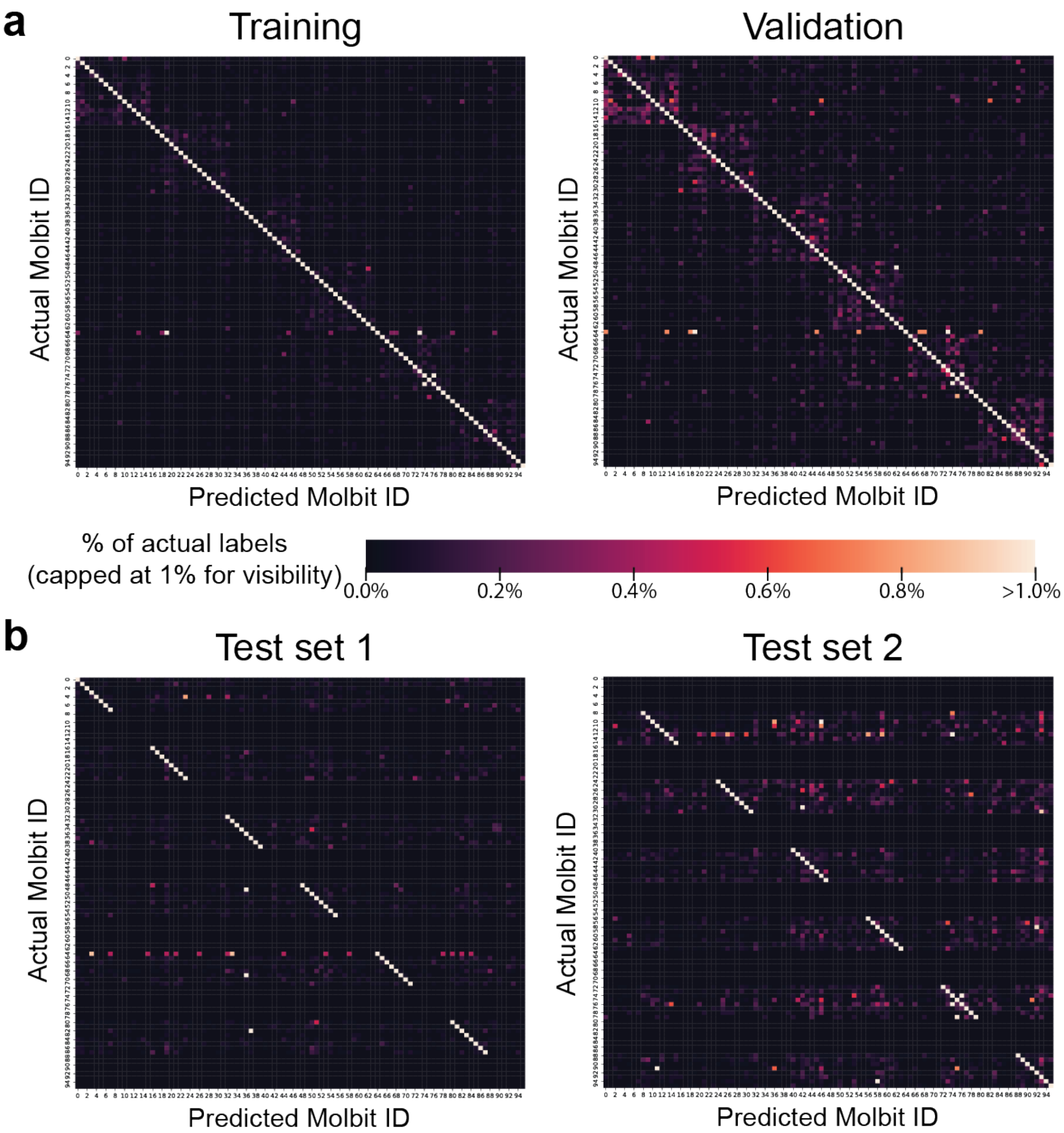

Supplementary Figure 6: Confusion matrices for training, validating, and testing the molbit classification model. (a) Training and validation. Since counts vary drastically for each molbit, values are normalized by the total number of actual (i.e., labeled via basecalling plus alignment) molbits. Due to high overall accuracy, the visualization is capped at 1% to make error patterns more visible. In the validation plot, some batching bias is visible, demonstrated by six squares surrounding the diagonal identity line. The 16 molbits within each of these six boxes were sequenced together in the same run. (b) Testing. As in (a), values are normalized by the total number of actual molbits. Each test set consists of a mutually exclusive set of half of the molbits, in this case arranged arbitrarily in groups of 8, causing the horizontal banding in the matrices.

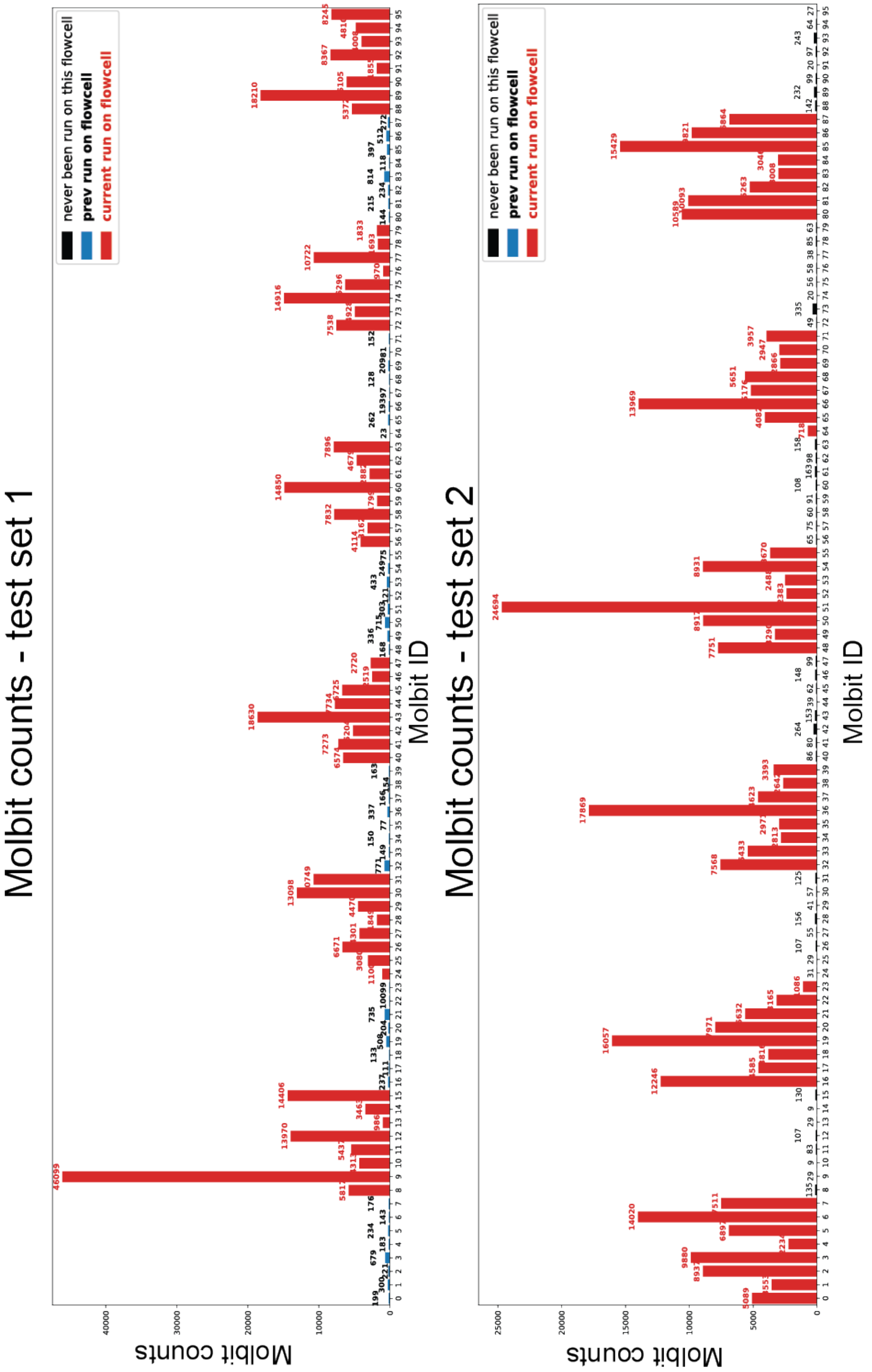

Supplementary Figure 7: Read counts per molbit for test runs.

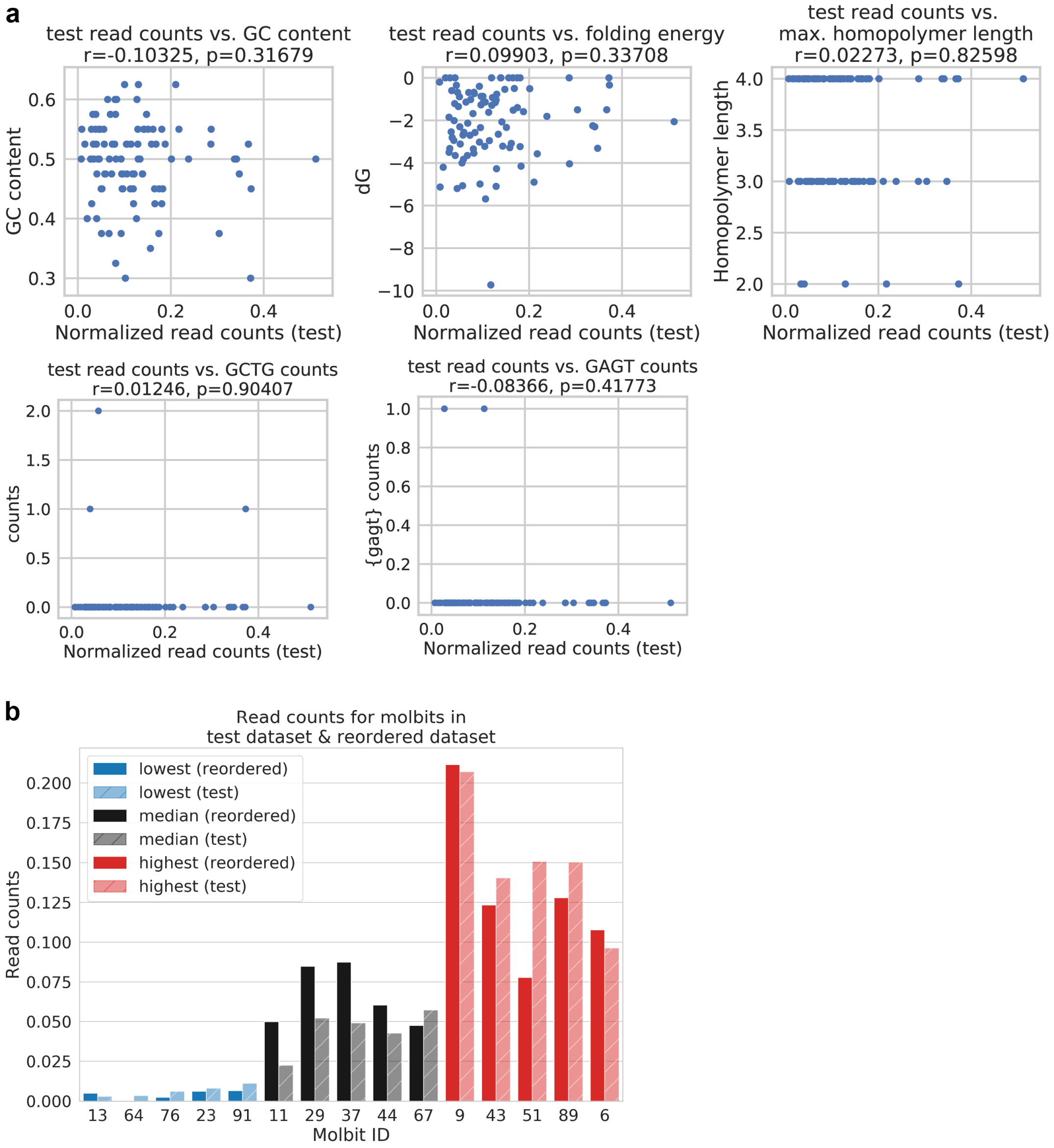

Supplementary Figure 8: Read count variation analysis. (a) Pearson correlation (two-sided) of normalized read counts against various sequence-related metrics: GC content, folding energy (δG minimum free energy), maximum homopolymer length, and mid-sequence presence of the assembly overhangs GCTG and GAGT). Normalized read counts were drawn from the two datasets used to test the model, and normalized training read counts showed similar trends. (b) Normalized read counts for molbit sequences that were reordered from IDT (solid bars), compared to the same molbits in the original test datasets (hashed bars). The reordered molbits were chosen randomly out of the lowest (blue), near-median (black), and highest (red) represented molbits.

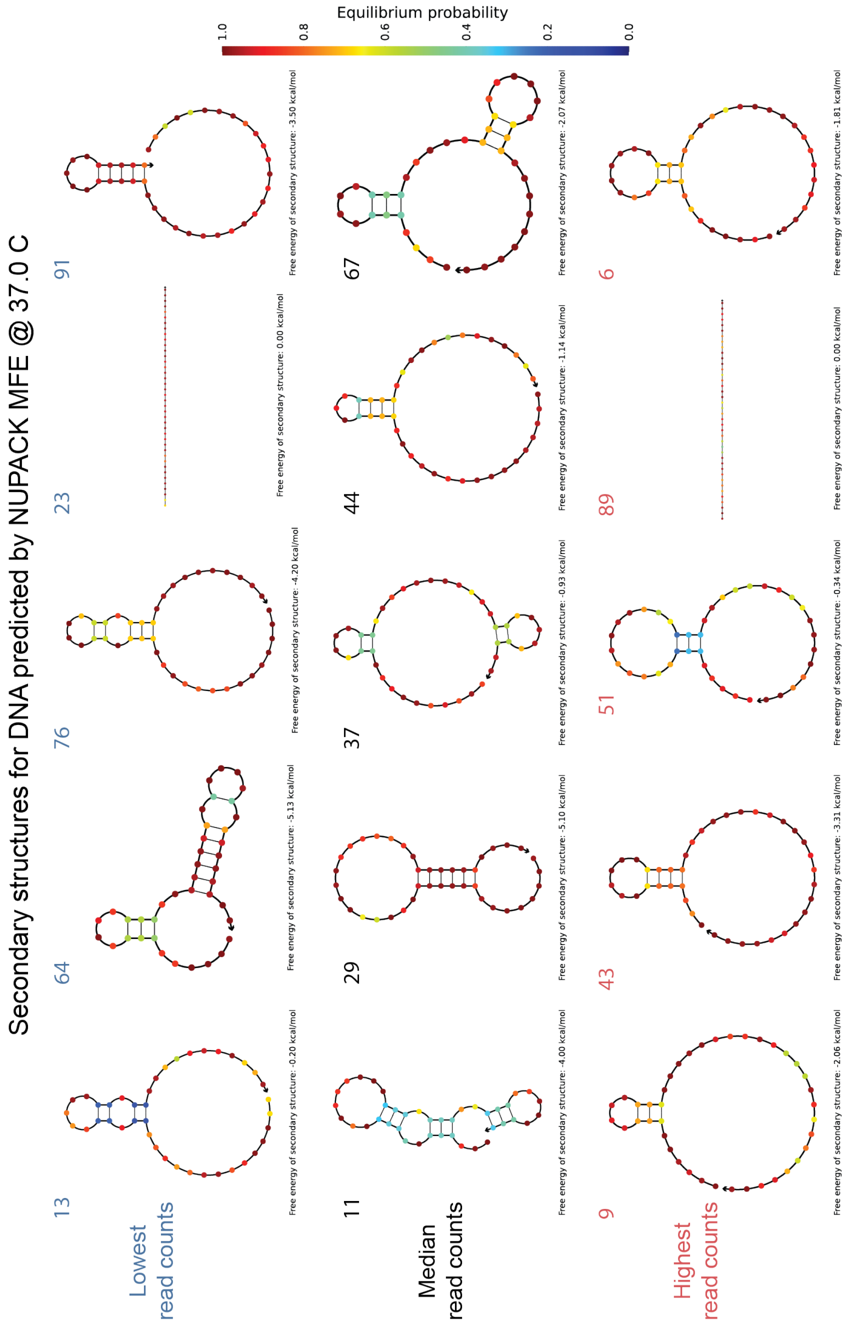

Supplementary Figure 9: NUPACK predicted folding and minimum free energy for re-ordered sequences. Top row contains low read count sequences, middle row contains average read count sequences, and bottom row contains high read count sequences.

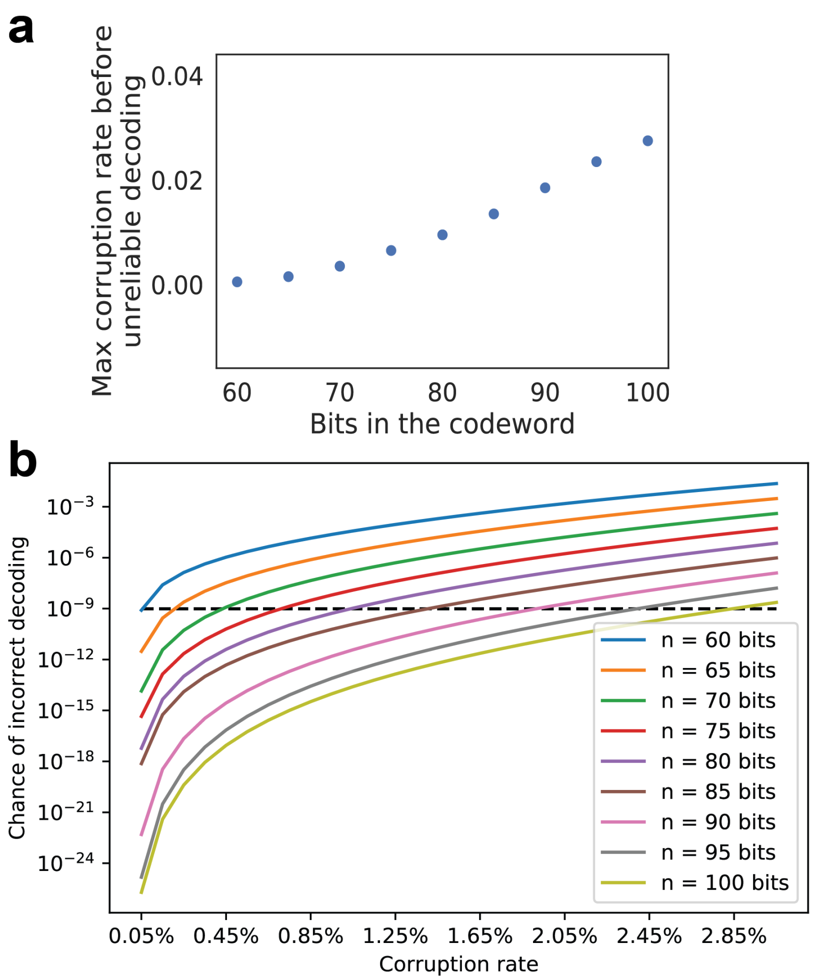

Supplementary Figure 10: Impact of bits in the codeword on the required corruption rates. (a) Bits in the codeword (n) vs. maximum corruption rate (k=32). Each point reflects the maximum corruption rate before the chance of incorrect decoding exceeds 10^{-9}. (b) Corruption rate vs the chance of incorrect decoding for different choices of n, (k=32). The dashed line is our desired incorrect decoding threshold; we want to stay below this line.

Supplementary References

[1] Krishnakumar, R. et al. Systematic and stochastic influences on the performance of the MinION nanopore sequencer across a range of nucleotide bias. Scientific Reports **8** (2018).

[2] Wick, R. R., Judd, L. M. & Holt, K. E. Performance of neural network basecalling tools for Oxford Nanopore sequencing. Genome Biology **20** (2019).

[3] Li, H. Aligning sequence reads, clone sequences and assembly contigs with BWA-MEM (2013). URL `http://arxiv.org/abs/1303.3997`.
