# Supplementary material for: Rapid and robust assembly and decoding of molecular tags with DNA-based nanopore signatures
Source: Nat Commun. 2020 Nov 3;11:5454. doi: 10.1038/s41467-020-19151-8 (PMC7642340; doi:10.1038/s41467-020-19151-8)
Supplement: Supplementary file 4 — Description of Additional Supplementary Files [file 41467_2020_19151_MOESM4_ESM.pdf]

Title: Supplementary Data 1

Description: Contains the molbit sequences in FASTA format. This is also included in the GitHub repository, but was added to the submission directly for completeness.
