# Supplementary material for: Rapid and robust assembly and decoding of molecular tags with DNA-based nanopore signatures
Source: Nat Commun. 2020 Nov 3;11:5454. doi: 10.1038/s41467-020-19151-8 (PMC7642340; doi:10.1038/s41467-020-19151-8)
Supplement: Supplementary file 5 — Reporting Summary [file 41467_2020_19151_MOESM5_ESM.pdf]

nature research

Corresponding author(s): Kathryn Doroschak, Jeff Nivala

Last updated by author(s): Apr 6, 2020

Reporting Summary

Nature Research wishes to improve the reproducibility of the work that we publish. This form provides structure for consistency and transparency in reporting. For further information on Nature Research policies, see Authors & Referees and the Editorial Policy Checklist .

Statistics

For all statistical analyses, confirm that the following items are present in the figure legend, table legend, main text, or Methods section.

n/a	Confirmed	
☐	☒	The exact sample size (*n*) for each experimental group/condition, given as a discrete number and unit of measurement
☐	☒	A statement on whether measurements were taken from distinct samples or whether the same sample was measured repeatedly
☐	☒	The statistical test(s) used AND whether they are one- or two-sided<br>*Only common tests should be described solely by name; describe more complex techniques in the Methods section.*
☐	☒	A description of all covariates tested
☐	☒	A description of any assumptions or corrections, such as tests of normality and adjustment for multiple comparisons
☐	☒	A full description of the statistical parameters including central tendency (e.g. means) or other basic estimates (e.g. regression coefficient) AND variation (e.g. standard deviation) or associated estimates of uncertainty (e.g. confidence intervals)
☒	☐	For null hypothesis testing, the test statistic (e.g. *F*, *t*, *r*) with confidence intervals, effect sizes, degrees of freedom and *P* value noted<br>*Give P values as exact values whenever suitable.*
☒	☐	For Bayesian analysis, information on the choice of priors and Markov chain Monte Carlo settings
☒	☐	For hierarchical and complex designs, identification of the appropriate level for tests and full reporting of outcomes
☐	☒	Estimates of effect sizes (e.g. Cohen's *d*, Pearson's *r*), indicating how they were calculated

Our web collection on statistics for biologists contains articles on many of the points above.

Software and code

Policy information about availability of computer code

Data collection	Oxford Nanopore Technologies' MinKNOW software (19.06.8) was used to acquire data from a MinION device.
Data analysis	Custom code was used to analyze the data (https://github.com/uwmisl/Porcupine), which includes code for designing molbits (including a custom SW implementation), identifying molbits from raw data, and scripts for analyzing and visualizing experimental results. Third party tools include Oxford Nanopore Technologies' Scrappie (1.3.2), Guppy (GPU 3.2.2), pytorch 1.1.0, CUDA 10, and UCR DTW. The following common python libraries were also used: seaborn, matplotlib, h5py, pandas, numpy, and jupyter.

For manuscripts utilizing custom algorithms or software that are central to the research but not yet described in published literature, software must be made available to editors/reviewers. We strongly encourage code deposition in a community repository (e.g. GitHub). See the Nature Research guidelines for submitting code & software for further information.

Data

Policy information about availability of data

All manuscripts must include a data availability statement. This statement should provide the following information, where applicable:

- Accession codes, unique identifiers, or web links for publicly available datasets
- A list of figures that have associated raw data
- A description of any restrictions on data availability

The FAST5 datasets generated and analyzed during the current study are available in the Harvard Dataverse repository (https://doi.org/10.7910/DVN/RCLFNB).

Field-specific reporting

Please select the one below that is the best fit for your research. If you are not sure, read the appropriate sections before making your selection.

Life sciences study design

All studies must disclose on these points even when the disclosure is negative.

Sample size	In this study, the sample size can be defined as the number of reads acquired per molecular bit for training data. This was chosen to balance training classes, and thus was limited by the minimum number of reads observed across all molbits. The sufficiency of the sample size can be approximated by overfitting in the machine learning trained to classify them, which did not seem to be an issue given the performance of the model on unseen test examples.
Data exclusions	Datasets were only excluded in the event of unsuccessfully completed protocols or failed sequencing.
Replication	Successfully encoding and decoding four previously-unseen molecular tags verified reproducibility of both the molecular bit detection model (CNN) and the overall decoding process including error correction.
Randomization	For gathering training data, the molecular bits were assigned numbered labels and then binned into batches based on their label.
Blinding	When encoding and decoding test molecular tags, the researcher running the computational software was typically kept unaware of the bits included in the tag, to avoid cherry-picking and other bias when dcoding.

Reporting for specific materials, systems and methods

We require information from authors about some types of materials, experimental systems and methods used in many studies. Here, indicate whether each material, system or method listed is relevant to your study. If you are not sure if a list item applies to your research, read the appropriate section before selecting a response.

Materials & experimental systems

n/a	Involved in the study	
☒	☐	Antibodies
☒	☐	Eukaryotic cell lines
☒	☐	Palaeontology
☒	☐	Animals and other organisms
☒	☐	Human research participants
☒	☐	Clinical data

Methods

n/a	Involved in the study	
☒	☐	ChIP-seq
☒	☐	Flow cytometry
☒	☐	MRI-based neuroimaging
